# Relationship between insulin sensitivity and gene expression in human skeletal muscle

**DOI:** 10.1186/s12902-021-00687-9

**Published:** 2021-02-27

**Authors:** Hemang M. Parikh, Targ Elgzyri, Amra Alibegovic, Natalie Hiscock, Ola Ekström, Karl-Fredrik Eriksson, Allan Vaag, Leif C. Groop, Kristoffer Ström, Ola Hansson

**Affiliations:** 1 Health Informatics Institute, Morsani College of Medicine, University of South Florida, 3650 Spectrum Blvd, Tampa, FL 33612 USA; 2 Department of Clinical Sciences, Diabetes & Endocrinology, Lund University, University Hospital Malmö, SE-20502 Malmö, Sweden; 3 Steno Diabetes Center, DK-2820 Gentofte, Denmark; 4 Unilever Discover R & D, Colworth Science Park, Sharnbrook, Bedfordshire, MK44 1LQ UK; 5 Finnish Institute of Molecular Medicine, FI-00014, University of Helsinki, Helsinki, Finland; 6 Swedish Winter Sports Research Centre, Mid Sweden University, SE-83125 Östersund, Sweden

## Abstract

**Background:**

Insulin resistance (IR) in skeletal muscle is a key feature of the pre-diabetic state, hypertension, dyslipidemia, cardiovascular diseases and also predicts type 2 diabetes. However, the underlying molecular mechanisms are still poorly understood.

**Methods:**

To explore these mechanisms, we related global skeletal muscle gene expression profiling of 38 non-diabetic men to a surrogate measure of insulin sensitivity, *i.e.* homeostatic model assessment of insulin resistance (HOMA-IR).

**Results:**

We identified 70 genes positively and 110 genes inversely correlated with insulin sensitivity in human skeletal muscle, identifying autophagy-related genes as positively correlated with insulin sensitivity. Replication in an independent study of 9 non-diabetic men resulted in 10 overlapping genes that strongly correlated with insulin sensitivity, including *SIRT2*, involved in lipid metabolism, and *FBXW5* that regulates mammalian target-of-rapamycin (mTOR) and autophagy. The expressions of *SIRT2* and *FBXW5* were also positively correlated with the expression of key genes promoting the phenotype of an insulin sensitive myocyte *e.g.* *PPARGC1A*.

**Conclusions:**

The muscle expression of 180 genes were correlated with insulin sensitivity. These data suggest that activation of genes involved in lipid metabolism, *e.g.* *SIRT2*, and genes regulating autophagy and mTOR signaling, *e.g.* *FBXW5*, are associated with increased insulin sensitivity in human skeletal muscle, reflecting a highly flexible nutrient sensing.

**Supplementary Information:**

The online version contains supplementary material available at 10.1186/s12902-021-00687-9.

## Background

Insulin resistance (or low insulin sensitivity) in skeletal muscle is a key feature of the pre-diabetic state and a predictor of type 2 diabetes (T2D) [1, 2]. It is also observed in individuals with hypertension, dyslipidemia, and cardiovascular diseases [3]. Insulin resistance (IR) in skeletal muscle has been attributed to different pathological conditions such as mitochondrial dysfunction [4], impaired glycogen synthesis [5], and accumulation of diacylglycerol with subsequent impairment of insulin signaling [6]. One hypothesis that has been put forward is a re-distribution of lipid stores from adipose tissue to non-adipose tissues (*e.g.* skeletal muscle, liver and the insulin-producing β-cells), the so-called overflow or ectopic fat distribution hypothesis. In support, studies have reported a strong correlation between intramuscular triacylglycerol (IMTG) content and IR [7, 8]. However, and in contrast, endurance-trained athletes have been shown to be highly insulin sensitive despite having large IMTG depots [9, 10]. One possible explanation for this discrepancy is that it is not the IMTG content per se that is important for the development of IR, but rather the relationship between IMTG content and muscle oxidative capacity. A reduced oxidative capacity in skeletal muscle from T2D individuals [11, 12], and in lean, insulin resistant offspring of T2D patients [13] has been found, supporting the hypothesis that IR in skeletal muscle is associated with dysregulation of intramyocellular fatty acid metabolism. Interestingly in a cohort of elderly twins, IMTG content seems to have a greater influence on hepatic as opposed to peripheral IR [14]. Furthermore, an association between mitochondrial dysfunction and decreased expression of autophagy-related genes in skeletal muscle from severely insulin resistant patients with T2D has previously been shown [15]. Conversely, enhancing autophagy in mice leads to an anti-ageing phenotype, including leanness and increased insulin sensitivity [16].

The aim of this study was therefore to investigate molecular mechanisms, *e.g.* IMTG content, associated with insulin sensitivity in skeletal muscle by relating global skeletal muscle gene expression with a surrogate measure of insulin sensitivity, *i.e.* homeostatic model assessment of insulin resistance (HOMA-IR).

## Methods

### Human participants and clinical measurements

Results from two separate clinical studies (studies A and B) are reported here.

### Study A

To identify genes correlated to insulin sensitivity in skeletal muscle, we studied 39 non-diabetic men from Malmö, Sweden [17, 18]. Briefly, the Malmö Exercise Intervention cohort consists of 50 sedentary but otherwise healthy male subjects from southern Sweden. They all have European ancestry and 24 of them have a first-degree family member with T2D. Muscle biopsies were collected from 39 of the subjects. The mean age and body mass index (BMI) were 37.71 ± 4.38 years and 28.47 ± 2.96 kg/m^2^, respectively, and the mean 1/the homeostatic model assessment-insulin resistance (HOMA-IR) was 0.69 ± 0.25 (Supplementary Table S1).

### Study B

To replicate the findings from study A, we studied an additional 10 healthy young non-diabetic men without any family history of diabetes, from a previously described study [19]. The mean age and BMI were 25.33 ± 0.99 years and 24.57 ± 1.86 kg/m^2^, respectively, and the mean 1/HOMA-IR was 1.17 ± 0.36 (Supplementary Table S2). Here, we included baseline gene expression profile data (*i.e.* only before bed rest) from part of a larger study on the influence of physical inactivity in healthy and prediabetic individuals [19].

None of the study participants were directed to avoid extreme physical exercise and alcohol intake for at least 2 days before the studies [20]. The participants were asked to fast for 10–12 h before examination days. Fasting blood samples and anthropometric data were obtained from all participants. All participants underwent an oral glucose tolerance test (OGTT; 75 g) and glucose tolerance was classified in accordance with World Health Organization criteria [21]. Homeostasis model assessment -insulin resistance (1/HOMA-IR = 22.5 / (fasting plasma insulin (μU/ml) x fasting plasma glucose (mmol/l))) was calculated for all participants in both studies and used as a surrogate measure of insulin sensitivity [22, 23]. The muscle biopsies were obtained from the *vastus lateralis* muscle under local anesthesia in individuals participating in all studies using a modified Bergström needle [24, 25].

We excluded data from two participants (one from each studies A and B) with extreme values of insulin sensitivity (more than 1.5 * interquartile range) for further analysis. Both studies were approved by local ethics committees and all participants gave their informed consent for participation.

### RNA extraction and hybridization

Muscle biopsies were taken from the right *vastus lateralis* muscle under local anesthesia (Lidocaine 1%), using a 6 mm Bergström needle (Stille AB, Sweden). In both studies, biopsies were immediately stored in RNAlater (Ambion, Austin, TX) and after overnight incubation at 4 °C snap frozen at − 80 °C until further processing. The double staining method was used for capillary staining. Myofibrillar ATPase histochemistry was performed by preincubation at pH 4.4, 4.6, and 10.3 to identify muscle fiber types [18]. Computer image analysis was performed using BioPix IQ 2.0.16 software (BioPix AB, Sweden). RNA was extracted using Tri reagent (Sigma-Aldrich, St. Louis, MO) followed by RNeasy Midi kit (Qiagen, Düsseldorf, Germany). The RNA was further concentrated by RNeasy MiniElute (Qiagen, Düsseldorf, Germany) and SpeedVac (DNA 120 SpeedVac, Thermo Savant, Waltham, MA).

For study A, synthesis of biotin-labeled cRNA and hybridization to the Affymetrix Custom Array NuGO-Hs1a520180 GeneChip (http://www.nugo.org) were performed according to the manufacturer’s recommendation. This GeneChip contains 23,941 probesets for interrogation, including known genes and expressed sequenced tags. Images were analyzed using the GeneChip Operating System (GCOS; Affymetrix) software. For each array, the percentage present call was greater than 40.

For study B, targets were hybridized to the one-color (Cy3, green) Agilent Whole Human Genome Oligo Microarray (G4112F (Feature Number version)) which contains 44,000 60-mer oligonucleotide probes representing 41,000 unique genes and transcripts. Probe labeling and hybridization were performed according to manufacturer’s recommendation. Images were analyzed using the Agilent Feature Extraction Software (version 9.5).

### Quantitative real-time PCR (QPCR)

A technical replication of the key findings from the microarray data, as well as expression analysis of key genes to be correlated with insulin stimulated glucose update, was conducted using QPCR. Reverse transcription was performed on 250 ng RNA (from 36 subjects in study A) or 200 ng RNA (from 7 subjects in the Muscle SATellite cell (MSAT) cohort) using the QuantiTect Reverse Transcription kit (Qiagen). QPCR was performed on a ViiA 7 real-time PCR system (Thermo Fisher Scientific) with 2 ng cDNA in 10 μl reactions and TaqMan Expression PCR Master Mix with duplex assays according to the manufacturer’s instructions. Samples were analyzed in triplicates on the same 384 well plate with 3 endogenous controls (*POL2A* (Hs00172187_m1), *HPRT1* (4326321E, VIC-MGB) and *PPIA* (4326316E, VIC-MGB)) for both studies A and B. The expression levels were calculated and normalized by geometric averaging of the endogenous controls as previously described [26]. Assays: *SIRT2* (Hs00247263_m1), *FBXW5* (Hs00382591_g1) and *CPT1B* (Hs00189258_m1). Endogenous control assays: *POLR2A* (Hs00172187_m1), *HPRT1* (4326321E, VIC-MGB) and *PPIA* (4326316E, VIC-MGB) for the 7 subjects in the Muscle SATellite cell (MSAT) cohort.

### Isolation and cultivation of human muscle satellite cells

Muscle satellite cells were isolated from 7 subjects from an ongoing unpublished MSAT study. Subjects were male with a mean age of 35.6 ± 10.6 years, a mean BMI of 25.1 ± 3.6 kg/m^2^ and a mean fasting plasma glucose value of 5.2 ± 0.2 mmol/L. Muscle biopsies were obtained from the *vastus lateralis* muscle under local anesthesia in individuals participating in all studies using a modified Bergström needle. Biopsies were minced into small pieces with scissors and digested in a digestion solution (Ham’s F-10 Nutrient mix (Gibco®, #31550015), Trypsin-EDTA (0.25%) (HyClone, SV30031.01), Collagenase IV (1 mg/ml) (Sigma, C5138), BSA (5 mg/ml) (Sigma, A2153)) at 37 °C for a total of 15–20 min. After this, cells were passed through a 70 μm cell strainer and centrifuged at 800 g for 7 min. The pellet was washed and resuspended in growth medium (Ham’s F-10 Nutrient Mix, GlutaMAX™ Supplement (Gibco®, #41550021), FBS (20%) (Sigma, F7524), Antibiotic/Antimycotic Solution (Gibco®, #15240062)) and cells were pre-plated on a culture dish and incubated for 3 h at 37 °C and 5% CO_2_ to allow fibroblast to attach to the plate. After this, the suspended cells were transferred to a flask pre-coated with matrigel (Corning #356234) and were incubated for 4 days at 37 °C and 5% CO_2_ in growth medium. Medium was then changed every other day. After about a week, cells were detached using TrypLE (TrypLE™ Express, no phenol red (Gibco®, #15090046)) and re-plated on the same flask to allow even distribution of cells over the surface.

At 70–80% confluence medium was changed first to an intermediate medium (DMEM, low glucose, GlutaMAX™ Supplement, pyruvate, No HEPES (Gibco® #21885025), FBS (10%) (Sigma, F7524), Antibiotics) for 24 h, and then to a differentiation medium (DMEM, low glucose, GlutaMAX™ Supplement, pyruvate, No HEPES (Gibco® #21885025), Horse serum (2%) (Invitrogen, #16050–130), Antibiotics) for 8 days, where glucose uptake experiments were performed. After 3 days of differentiation, Cytarabine (Ara-C) (10 μg/ml) (Sigma, C1768) was added to the differentiation medium, for 2 days, to prevent excessive growth of proliferating cells, *e.g.* fibroblasts [27].

### Measurement of glucose uptake in cultured muscle cells

Measurement of glucose uptake in cultured muscle cells was performed using an enzymatic fluorometric assay as previously described [28]. Briefly, cells differentiated for 8 days grown in 12-well plates, were starved for 3 h in FBS-free DMHG low glucose medium (Gibco® #21885025) at 37 °C and 5% CO_2_. The cells were then washed in warm PBS and treated with either Cytochalasin B (10 μM) (Sigma, C6762) (for non-specific glucose uptake), Krebs-Ringer-HEPES (KRH) buffer only (basal glucose uptake) or with 100 nM insulin (Actrapid 100 IE/ml, Novo Nordisk) (stimulated glucose uptake) in a KRH buffer containing 0.1% BSA (pH 7.4) for 1 h at 37 °C and 5% CO_2_. After this, cells were incubated in a KRH buffer containing 2-Deoxy-D-glucose (2DG) (1 mM) (Sigma, D6134) for 15 min at room temperature, after which the cells were washed in ice-cold PBS and then frozen and stored at − 80 °C (for less than a week). Lysis was done by adding 0.1 M ice cold NaOH to the cells and incubate at 70 °C for 60 min, after which HCL and triethanolamine (TEA) buffer (pH 8.1) (Sigma, T1502) at final concentrations of 0.1 M and 50 mM respectively, were added to neutralize the lysate. Lysates and prepared series 2-Deoxy-D-glucose 6-phosphate (DG6P) (Santa Cruz, SC-220734) dilution standards (30, 15, 7.5, 3.75, 1.875, 0 μM) (dissolved in “lysate buffer” (0.1 M NaOH / 0.1 M HCl / 50 mM TEA buffer, pH 8.1; same proportion as samples), were transferred to a black 96-well assay plate (Greiner Bio-one International, 655076), 250 μl of assay solution (TEA buffer (50 mM) with KCl (50 mM) (pH 8), BSA (0.02%), NADP (0.1 mM) (Sigma, N8035), Diaphorase (0.2 U/ml) (Sigma, D2197), Resazurin (6 μM) (Sigma, R7017), G6PDH (15.4 U/ml) (Sigma, G8404)) was added to each well, and the plates were incubated for 60 min at 37 °C. Fluorescence was measured using the microplate reader (Infinite M200 Pro, Tecan) at wavelengths λ_ex_ = 545 nm and λ_em_ = 590 nm. DG6P was then quantified by comparing the fluorescence intensity from the experimental samples to the DG6P standard curve. Value were adjusted for protein concentration determined with the Pierce™ Coomassie (Bradford) Protein Assay Kit (Thermo Fisher Scientific, 23200).

### Quantification of mtDNA content

DNA was isolated from the muscle biopsies by phenol/chloroform/isoamyl alcohol extraction according to the manufacturer’s recommendation (Diagenode, Belgium). Concentration and purity were measured using a NanoDrop ND-1000 spectrophotometer (A_260_/A_280_ > 1.6 and A_260_/A_230_ > 1.0) (NanoDrop Technologies, Wilmington, DE, USA). QPCR was carried out using an Applied Biosystems 7900HT sequence detection system with 5 ng genomic DNA in 10 μl reactions and TaqMan Expression PCR Master Mix according to the manufacturer’s recommendations. All samples were analyzed in triplicates on the same 384 well plate (maximum accepted standard deviation in Ct-value of 0.1 cycles). Two assays (*16S* and *ND6*) were used to analyze mitochondrial DNA content (mtDNA) targeting the heavy and light strand, respectively. To analyze nuclear DNA (nDNA) content *RNaseP* was used as a target. The mtDNA content is calculated as the mean value of *ND6* and *16S* divided by 2 x *RNaseP*. Assays used: *ND6* (Hs02596879_g1), *16S* (Hs02596860_s1) and *RNaseP* (4316838).

### Statistical analysis

#### Study A

We used ENTREZ custom chip definition files (http://brainarray.mbni.med.umich.edu) to regroup the individual probes into consistent probesets and remap to the correct sets of genes for Affymetrix Custom Array NuGO-Hs1a520180 array which resulted in a total of 16,313 genes from study A. We used three different procedures for normalization and summarization as described previously [29]: (1) The GC-content robust multi-array average (GC-RMA) method, (2) Probe logarithmic intensity error (PLIER) method (Affymetrix), and (3) Robust multi-array average (RMA) method [30–34]. We conducted filtering based on the Affymetrix microarray suite version 5.0 (MAS5.0) present/absent calls which classified each gene as expressed above background (present call) or not (absent or marginal call). We included genes, which have detection call as present call in at least 25% of arrays [35], which left 7947 genes out of 16,313 for further analysis in study A.

To identify a reliable list of genes regulating insulin sensitivity, Spearman partial correlation analysis was performed to determine the individual effects of each gene expression on a surrogate measure of insulin sensitivity (1/HOMA-IR) after adjusting for BMI, age and family history of T2D for each of three normalization methods namely GC-RMA, PLIER and RMA separately. We considered only those genes that were significantly correlated with insulin sensitivity with a *P* <  0.05 in all three different normalization methods.

To technically validate the microarray findings, real time quantitative PCR (QPCR) was used to measure the mRNA expression of *FBXW5* and *SIRT2* in human skeletal muscle from study A. Correlation between the microarray and QPCR experiments was determined using Spearman’s rank correlation coefficient test.

In the study A cohort, correlation between the QPCR expression values of *SIRT2*, *FBXW5*, *CPT1B*, *FABP3*, *MLYCD*, *PPARG1A* and *ESRRA* with % fiber type and mitochondrial DNA was determined using Spearman’s rank correlation coefficient test. All data except that of *SIRT2* and *FBXW5* was collected and reanalyzed from a previously described study [17, 18].

Enrichment analyses were performed on the genes whose expression levels in skeletal muscle were significantly correlated with insulin sensitivity in study A using the WEB-based GEne SeT AnaLysis Toolkit (WebGestalt) which implements the hypergeometric test [36].

#### Study B

The median intensities of each spot on the array were calculated using the GenePix Pro software (version 6). We performed quantile-based normalization between arrays without background subtraction using linear models for microarray data (limma) package in R [37, 38]. We removed poor quality probes that were either saturated (*i.e.* > 50% of the pixels in a feature are above the saturation threshold) or flagged as non-uniformity outlier (*i.e.* the pixel noise of feature exceeds a threshold for a uniform feature) in at least one array, which left 29,297 probes for further analysis [39].

Spearman partial correlation analysis was performed to determine the individual effects of each gene expression on a surrogate measure of insulin sensitivity (1/HOMA-IR) after adjusting for BMI and age. Due to the exploratory nature of the study, no correction for multiple testing was performed. Instead, we considered only those genes that were significantly, positively or inversely, correlated with insulin sensitivity in both studies A and B with a significance level set to 0.05. Paired Wilcoxon signed-rank test was conducted to assess for the change before and after insulin-stimulated glucose uptake. Spearman correlation analysis was between basal- and insulin-stimulated glucose uptake and mRNA expression of *FBXW5*, *SIRT2* and *CPT1B*. All statistical analyses were performed using IBM® SPSS® Statistics, MATLAB® and R statistical software. The microarray data both studies have been deposited in the National Center for Biotechnology Information’s Gene Expression Omnibus (GEO) database (http://www.ncbi.nlm.nih.gov/geo); series accession number is GSE161721.

## Results

To identify genes with skeletal muscle expression related to insulin sensitivity, we obtained muscle biopsies from 38 non-diabetic participants in study A (the data from one participant was excluded, Methods). Clinical characteristics of these participants are shown in Supplementary Table S1. We then profiled muscle gene expression using Affymetrix oligonucleotide microarrays. To replicate the findings from study A, we included 9 non-diabetic participants from study B (the data from one participant was excluded, Methods). Clinical characteristics of these participants are shown in Supplementary Table S2. We performed skeletal muscle gene expression profiling from these participants using the Agilent oligonucleotide microarrays. Insulin sensitivity was estimated using the 1/HOMA-IR method calculated from OGTT values (Methods).

### Correlation with insulin sensitivity

#### Study A

We identified 70 genes positively (Supplementary Table S3) and 110 genes inversely (Supplementary Table S4) correlated with insulin sensitivity in human skeletal muscle. Using WebGestalt [36], we performed enrichment analyses of genes significantly correlated to insulin sensitivity. Of the Gene Ontology (GO) categories overrepresented in the 70 genes positively correlated to insulin sensitivity (Supplementary Table S3), several were related to autophagy (Supplementary Table S5). Among enriched Wikipathways of the positively correlated genes were mTOR signaling and thermogenesis (Supplementary Table S6). Enriched GO categories of the genes inversely correlated to insulin sensitivity (Supplementary Table S4) included platelet-derived growth factor binding, fibrillar collagen trimer, banded collagen fibril and complex of collagen trimers (Supplementary Table S7).

**Fig. 1:**
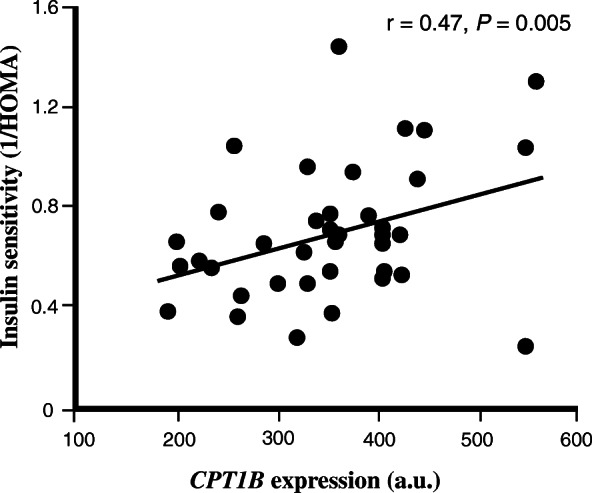
The skeletal muscle expression of *CPT1B* correlates with insulin sensitivity (1/HOMA-IR) in study A. The relationship between insulin sensitivity (1/HOMA-IR) and skeletal muscle expression of *CPT1B* in study A (*n* = 38). For illustration, gene expression data is shown for only GC-content robust multi-array average (GC-RMA) method

Among genes positively correlated with insulin sensitivity, several, including *F-box and WD repeat domain containing 5* (*FBXW5*), *TSC2*, *ULK1*, *ATG13, AKT1S1, SQSTM1* and *TFEB* were found to be regulated by or regulating mammalian target-of-rapamycin (mTOR) signaling and autophagy. Among genes involved in lipid metabolism were *carnitine palmitoyltransferase 1B* (*CPT1B*) (Fig. 1), the rate limiting enzyme for fatty acid oxidation, *SLC27A1* (also known as *long chain-fatty acid transport protein 1*), a major transporter of fatty acids across the plasma membrane and *PNPLA2* (also known as *adipocyte triglyceride lipase* (*ATGL*)) a triglyceride lipase known to be expressed in human skeletal muscle [40]. Also, the *sirtuin 2* (*SIRT2*) gene positively correlated with insulin sensitivity, which is a family member of SIRT1 with well-known effects on peripheral insulin signaling [41]. Other interesting genes with relevance for skeletal muscle insulin sensitivity were *uncoupling protein 2* (*UCP2),* an inner mitochondrial membrane protein, and genes with direct functional roles in skeletal muscle, *e.g. obscurin*, *histidine rich calcium binding protein* (*HCR*) and *myocyte enhancer factor 2D* (*MEF2D*) (Supplementary Table S3).

Among the genes inversely correlated with insulin sensitivity, several were associated with the extracellular matrix, such as *collagen type I alpha 1 chain* (*COL1A1*), *collagen type I alpha 2 chain* (*COL1A2*), *collagen type III alpha 1 chain* (*COL3A1*) and *laminin subunit alpha 4* (*LAMA4*) (Supplementary Table S4).

#### Study B

In order to replicate the findings in study A, we analyzed muscle expression in an additional 9 healthy young non-diabetic men without any family history of diabetes. Of the genes found to be correlated with insulin sensitivity, 10 were replicated in study B. Seven of these genes were positively correlated (*SIRT2*, *FBXW5*, *RAB11FIP5*, *CPT1B*, *C16orf86*, *UCKL1* and *ARFGAP2*) and three were inversely correlated (*ZNF613*, *UTP6* and *LEO1*) with insulin sensitivity (Table 1).

**Table 1:** Genes of which the expression levels in skeletal muscle were correlated with insulin sensitivity in both study A and B. Spearman partial correlation analysis was performed to determine the individual effects of each gene expression on a surrogate measure of insulin sensitivity (1/HOMA-IR). Significance level was set to 0.05

Gene Symbol	Entrez GeneID	Study A (GC-RMA)		Study B	
		r	***P***	r	***P***
*SIRT2*	22933	0.39	0.021	0.95	0.001
*FBXW5*	54461	0.35	0.041	0.95	0.001
*RAB11FIP5*	26056	0.38	0.023	0.93	0.002
*CPT1B*	1375	0.47	0.005	0.85	0.015
*ZNF613*	79898	−0.34	0.047	−0.81	0.027
*UTP6*	55813	− 0.36	0.033	− 0.81	0.028
*C16orf86*	388284	0.46	0.005	0.79	0.036
*LEO1*	123169	−0.43	0.010	−0.78	0.039
*UCKL1*	54963	0.47	0.004	0.77	0.044
*ARFGAP2*	84364	0.38	0.024	0.76	0.047

*Abbreviations*: GC-RMA, GC-content robust multi-array average; r, Spearman partial correlation coefficient

Results are ranked based on *P* from Study B

### Technical validation of the microarray data using real time quantitative PCR (QPCR)

**Fig. 2:**
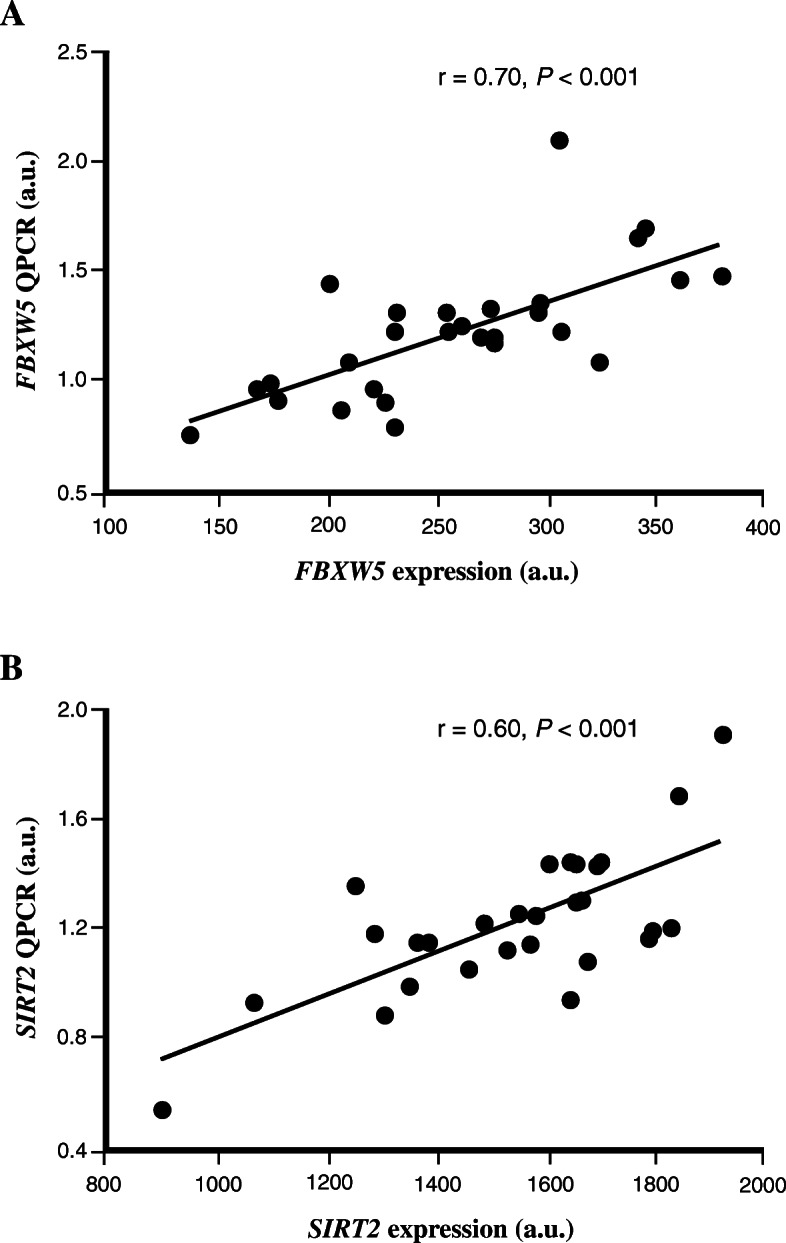
Technical replication of the microarray data using QPCR. The relative expression level of (**a**) *FBXW5* and (**b**) *SIRT2* genes were measured in study A using both microarray (x-axis) and QPCR (y-axis). Data was analyzed with Spearman’s rank correlation coefficient test (*n* = 29). For illustration, gene expression data is shown for only GC-content robust multi-array average (GC-RMA) method

To technically validate the microarray findings, QPCR was used to measure the mRNA expression of *FBXW5* and *SIRT2* in human skeletal muscle from study A. Significant correlation between the microarray and QPCR experiments was observed for both *FBXW5* (r = 0.70, *P* <  0.001), *SIRT2* (r = 0.60, *P* <  0.001) (Fig. 2) and *CPT1B* (r = 0.74, *P* <  0.001) (previously shown [18]). The expression of *FBXW5* and *SIRT2* analyzed with QPCR was also positively correlated with each other (r = 0.81, *P* <  0.001) and with the QPCR expression value of *CPT1B* (Table 2).

**Table 2:** Correlation between the gene expression of *SIRT2*, *FBXW5* and *CPT1B* analyzed with quantitative PCR (QPCR) with the QPCR expression values of *SIRT2*, *FBXW5*, *CPT1B*, key genes involved in transport and mitochondrial uptake and oxidation of fatty acids in skeletal muscle (*FABP3* and *MLYCD*) and genes with central roles in regulating mitochondrial biogenesis and oxidative phosphorylation in muscle (*PPARGC1A* and *ESRRA*). Significant correlation was determined using Spearman’s rank correlation coefficient test

		***SIRT2***	***FBXW5***	***CPT1B***
***SIRT2***	Spearman’s rank correlation coefficient	–	0.81	0.71
	*P*	–	< 0.001	< 0.001
	n	–	36	35
***FBXW5***	Spearman’s rank correlation coefficient	0.81	–	0.60
	*P*	< 0.001	–	< 0.001
	n	36	–	35
***CPT1B***	Spearman’s rank correlation coefficient	0.71	0.60	–
	*P*	< 0.001	< 0.001	–
	n	35	35	–
***FABP3***	Spearman’s rank correlation coefficient	0.54	0.49	0.75
	*P*	0.001	0.003	< 0.001
	n	35	35	37
***MLYCD***	Spearman’s rank correlation coefficient	0.66	0.59	0.76
	*P*	< 0.001	< 0.001	< 0.001
	n	35	35	37
***PPARGC1A***	Spearman’s rank correlation coefficient	0.44	0.46	0.66
	*P*	0.009	0.005	< 0.001
	n	35	35	37
***ESRRA***	Spearman’s rank correlation coefficient	0.67	0.66	0.69
	*P*	< 0.001	< 0.001	< 0.001
	n	32	32	32

*Abbreviations*: *SIRT2*, sirtuin 2; *FBXW5*, F-box and WD repeat domain containing 5; *CPT1B*, carnitine palmitoyltransferase 1B; *PPARGC1A* (*PGC1α*), peroxisome proliferator-activated receptor gamma coactivator 1-alpha; *ESRRA*, estrogen-related receptor alpha; *MLYCD*, malonyl-CoA decarboxylase; *FABP3*, fatty acid binding protein 3

### Correlation between the QPCR expression of the replicated genes *FBXW5*, *SIRT2* and *CPT1B* with the expression of key metabolic genes, fiber type and mitochondrial DNA content in skeletal muscle from study A participants and with in vitro glucose uptake in human myotube cells

The expression of *FBXW5*, *SIRT2* and *CPT1B*, was positively correlated with *malonyl-CoA decarboxylase* (*MLYCD*) and *fatty acid binding protein 3* (*FABP3*)*,* key genes involved in transport and mitochondrial uptake and oxidation of fatty acids in muscle, and with *estrogen related receptor alpha* (*ESRRA*) and *PPARGC1A* (also known as *PGC1α*) (Table 2), *i.e.* with genes playing central roles in regulating mitochondrial biogenesis and oxidative phosphorylation in muscle [42]. Also, expression of *FBXW5*, *SIRT2* and *CPT1B* was positively correlated with percent type I and inversely correlated with percent type II B fibers in skeletal muscle, and the expression of *SIRT2* and *CPT1B* was also positively correlated with the amount of mitochondrial DNA (Table 3).

**Table 3:** Correlation between the gene expression of *SIRT2*, *FBXW5* and *CPT1B* analyzed with quantitative PCR (QPCR) with % fiber type and mitochondrial DNA. Significant correlation was determined using Spearman’s rank correlation coefficient test

		***SIRT2***	***FBXW5***	***CPT1B***
**% type I fiber**	Spearman’s rank correlation coefficient	0.46	0.47	0.44
	*P*	0.006	0.005	0.008
	n	34	34	35
**% type II A fiber**	Spearman’s rank correlation coefficient	0.20	0.03	0.19
	*P*	0.27	0.88	0.31
	n	31	31	32
**% type II B fiber**	Spearman’s rank correlation coefficient	−0.53	−0.46	−0.46
	*P*	0.002	0.01	0.007
	n	31	31	32
**Mitochondrial DNA**	Spearman’s rank correlation coefficient	0.42	0.25	0.40
	*P*	0.013	0.15	0.017
	n	34	34	35

*Abbreviations*: *SIRT2*, sirtuin 2; *FBXW5*, F-box and WD repeat domain containing 5; *CPT1B*, carnitine palmitoyltransferase 1B

**Fig. 3:**
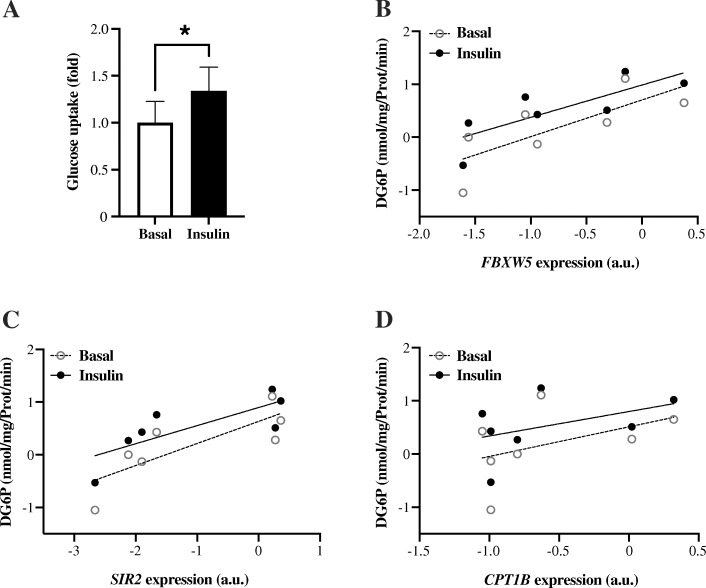
Glucose uptake in isolated human myotube cells. **a** Insulin-stimulated glucose uptake: Insulin stimulation (1 h) led to a ~ 35% increase (*P* = 0.018) in glucose uptake. Correlation between basal- and insulin-stimulated glucose uptake and mRNA expression of (**b**) *FBXW5* (r_basal_ = 0.79, *P* = 0.036 and r_insulin_ = 0.86, *P* = 0.014) (**c**) *SIRT2* (r_basal_ = 0.79, *P* = 0.036 and r_insulin_ = 0.82, *P* = 0.023) and (**d**) *CPT1B* (r_basal_ = 0.49, *P* = 0.268 and r_insulin_ = 0.43, *P* = 0.333). *n* = 7 average values from 1 to 3 independent experiments per individual. For illustration, data were transformed using the natural logarithm (**b**-**d**)

From the Muscle SATellite cell (MSAT) study, skeletal muscle myoblast cells were isolated and differentiated to myotubes. Insulin stimulation of the myotubes led to a ~ 35% increase (*P* = 0.018) in glucose uptake (Fig. 3a). Expression of *FBXW5* (r = 0.79, *P* = 0.036) and *SIRT2* (r = 0.79, *P* = 0.036), but not *CPT1B* (r = 0.49, *P* = 0.268) were positively correlated with glucose uptake during the basal, non-insulin-stimulated state (Fig. 3b-d). Similar correlations were observed in the insulin-stimulated state, *i.e.* *FBXW5* (r = 0.86, *P* = 0.014) and *SIRT2* (r = 0.82, *P* = 0.023), but not for *CPT1B* (r = 0.43, *P* = 0.333) (Fig. 3b-d).

## Discussion

The objective of this study was to identify genes for which expression levels are correlated with insulin sensitivity in human skeletal muscle. Genes involved in fatty acid metabolism (*CPT1B* and *SIRT2*) and in autophagy and mTOR signaling (*FBXW5*), *TSC complex subunit 2* (*TSC2*) and *unc-51 like autophagy activating kinase 1* (*ULK1*) were found to be associated with insulin sensitivity and related traits (muscle fiber type distribution and mitochondrial number).

We replicated the findings for 10 genes from Study A in Study B using Agilent oligonucleotide microarrays, consisting of 60-mers probes compared to the short 25-mers probes utilized by Affymetrix. The expressions of *SIRT2*, *FBXW5*, *RAB11FIP5*, *CPT1B*, *C16orf86*, *UCKL1* and *ARFGAP2* were positively, whereas the expressions of *ZNF613*, *UTP6* and *LEO1* were inversely correlated with insulin sensitivity as assessed by 1/HOMA-IR in both studies.

Among the replicated genes positively correlated with insulin sensitivity was *CPT1B*. CPT1B regulates the transport of long-chain fatty acyl-CoAs from the cytoplasm into the mitochondria, a key regulatory step in lipid β-oxidation. There is strong evidence that β-oxidation, plays a crucial role in the development of IR, where inhibition of *Cpt1b* induces [43] and overexpression of *Cpt1b* ameliorates [44] IR in rats. Also, a common haplotype of *CPT1B* has been associated with the metabolic syndrome in male participants [45]. The krüppel-like transcription factor (KLF5) together with C/EBP-β and PPARδ regulate the expression of *CPT1B* and *UCP2* (also positively correlated with insulin sensitivity (Supplementary Table S3) in skeletal muscle) [46]. Moreover, expression of *Cpt1b* and *Ucp2* in skeletal muscle is up-regulated in the klf5-knockout heterozygous mouse, which is resistant to high fat-induced obesity and glucose intolerance. The skeletal muscle expression of *CPT1B* in humans is increased after treatment with a PPARδ agonist [47], and this agonist is also shown to increase muscle mitochondrial biogenesis and improve glucose homeostasis, the latter suggested to be mediated by enhanced fatty acid catabolism in muscle [48]. It is likely that the beneficial effect of the PPARδ agonist is partly due to induction of *CPT1B* in skeletal muscle. Other genes coupled to lipid metabolism whose expression positively correlated with insulin sensitivity include *PNPLA2* (*ATGL*) and *SLC27A1* (*long chain-fatty acid transport protein 1*; *FATP-1*). Although no correlation between insulin sensitivity and muscle *ATGL* expression has previously been reported, *ATGL* mRNA is shown to be strongly coupled to mRNA levels of *CPT1B* in human muscle [49]. *Atgl*, *Cpt1b* and *Slc27a1* are highly expressed in insulin responsive oxidative type I fibers, and insulin-stimulated fatty acid uptake is largely dependent on *Slc27a1* in rodent muscle [50]. Taken together, data presented here are in-line with and support previous findings that skeletal muscle lipid metabolism, and lipid β-oxidation in particular, plays an important role in the development of IR.

Another replicated gene in this study positively correlated with insulin sensitivity was *SIRT2*, a predominantly cytoplasmic deacetylase expressed in a wide range of metabolically relevant tissues. Increasing evidence suggests that the expression of *SIRT2* is modulated in response to energy availability, being induced during low-energy status [51]. Conversely, dietary obesity and associated pathologies, *e.g.* IR, is linked to the capacity to suppress β-oxidation in visceral adipocytes, in part through transcriptional repression of *SIRT2* with negative effects on the SIRT2-PGC1α regulatory axis [52]. SIRT2 is also described as a novel AKT interactor, critical for AKT activation by insulin, and the potential usefulness of *SIRT2* activators in the treatment of insulin-resistant metabolic disorders has been discussed [53]. Unlike the well-documented effects of SIRT1 in skeletal muscle insulin signaling [41], the role of SIRT2 in skeletal muscle is much less defined. A study using mouse C2C12 skeletal muscle cells showed that down-regulation of Sirt2 in insulin resistant cells improved insulin sensitivity [54], raising the possibility that Sirt2 has tissue-specific roles regarding insulin sensitivity. The opposite findings presented here, showing a positive association between insulin sensitivity and *SIRT2* gene expression in human skeletal muscle could highlight a differential role in various metabolic conditions, or species differences.

Of the enriched Gene Ontology (GO) categories of genes whose expression positively correlated with insulin sensitivity, several were related to autophagy, process utilizing autophagic mechanism and regulation of macroautophagy. Interestingly, we found the expression of *FBXW5* to be positively correlated to insulin sensitivity in both study A and B. FBXW5 is part of an E3 ubiquitin ligase that regulates TSC2 protein stability and complex turnover [55], with indirect effects on mTOR. Moreover, a variant (rs546064512) in *FBXW5* is shown to be associated with total cholesterol (odds ratio = 0.56 and *P* = 8.93 × 10^− 4^) in 12,940 individuals of multiple ancestries ([56] and The T2D Knowledge Portal: http://www.type2diabetesgenetics.org/). In the fed state insulin signaling activates mTOR, whereas in the fasted state AMPK has the opposite effect leading to inactivation of mTOR and activation of autophagy. ULK1 negatively regulates and is negatively regulated by mTOR, making mTOR a major convergence point for the regulation of autophagy [57]. ULK1 is also a key regulator of mitophagy, and it’s phosphorylation by AMPK is required for mitochondrial homeostasis and cell survival during starvation [58]. The large number of autophagy-related genes positively correlating with insulin sensitivity might result from the fasted state of the subjects (10–12 h) and could be a reflection of metabolic flexibility, *i.e.*, the ability to switch from high rates of fatty acid uptake and lipid oxidation to suppression of lipid metabolism with a paralleled increase in glucose uptake, storage and oxidation in response to, *e.g.*, feeding or exercise. Impaired autophagy has been implicated in ageing and IR, and induction of autophagy is required for muscle glucose homeostasis mediated by exercise in mice [59]. A crucial link between autophagy and insulin sensitivity in humans has been suggested in a study where skeletal muscle from severely insulin resistant subjects with T2D show a highly altered gene expression related to mitochondrial dysfunction and abnormal morphology, and that this is associated with decreased expression of autophagy-related genes [15].

Future studies are required to determine the potential role of the remaining replicated genes in the regulation of insulin sensitivity in human skeletal muscle, although it should be mentioned that RAB11FIP5, an AS160- and Rab-binding protein, is suggested to coordinate the protein kinase signaling and trafficking machinery required for insulin-stimulated glucose uptake in adipocytes [60]. Also, RAB11FIP5 is an effector protein of RAB11, a GTPase that regulates endosomal trafficking shown to be required for autophagosome formation [61], suggesting yet another link between the regulation of insulin sensitivity and autophagy in skeletal muscle.

The positive correlation of *CPT1B*, *SIRT2* and *FBXW5* expression with insulin sensitivity in this study is supported by the observed positive correlation of these genes with the expression of key genes promoting the phenotype of an insulin sensitive myocyte, *e.g.* transport and mitochondrial uptake and oxidation of fatty acids and positive regulation of mitochondrial biogenesis and oxidative phosphorylation (Tables 2 and 3). For *SIRT2* and *FBXW5*, this was also supported by the correlation of these genes with glucose uptake measurements in human myotube cells (Fig. 3).

There are several issues to consider in the interpretation of the results. In both studies, we used 1/HOMA-IR as a surrogate measure of insulin sensitivity. The HOMA-IR index is based upon fasting measurements of insulin and glucose and thus more reflects variation in hepatic than in peripheral insulin sensitivity [62]. Although several studies have shown significant correlations between HOMA-IR and insulin-stimulated glucose uptake as measured by an euglycemic hyperinsulinemic clamp, this correlation cannot be expected to be very strong given the different physiological conditions they reflect [22, 63]. On the other hand, biopsies in both studies were obtained in the fasting state and should thus more correspond to conditions as measured by 1/HOMA-IR.

## Conclusions

In conclusion, we present a catalog with muscle expression of 180 genes correlated with insulin sensitivity. This data provides compelling evidence that activation of genes involved in lipid metabolism, including *SIRT2*, and of genes involved the regulation of autophagy and mTOR signaling, *e.g.* *FBXW5*, are associated with increased insulin sensitivity in human skeletal muscle. Determining if these genes are causally related with insulin sensitivity in humans should be the aim of future studies.

## Supplementary Information

**Additional file 1: Supplementary Table S1.** Clinical and biochemical characteristics of male subjects from study A. **Supplementary Table S2.** Clinical and biochemical characteristics of male subjects from study B. **Supplementary Table S3.** Genes of which expression levels in skeletal muscle were positively correlated with insulin sensitivity (1/HOMA-IR) in study A. **Supplementary Table S4.** Genes of which expression levels in skeletal muscle were inversely correlated with insulin sensitivity (1/HOMA-IR) in study A. **Supplementary Table S5.** Significantly enriched Gene Ontology (GO) categories in the 70 genes whose expression level in skeletal muscle positively correlated with insulin sensitivity in Study A, analyzed with the WEB-based GEne SeT AnaLysis Toolkit (WebGestalt). **Supplementary Table S6.** Significantly enriched Wikipathways, in the 70 genes whose expression level in skeletal muscle positively correlated with insulin sensitivity in Study A, analyzed with the WEB-based GEne SeT AnaLysis Toolkit (WebGestalt). **Supplementary Table S7.** Significantly enriched Gene Ontology (GO) categories in the 110 genes whose expression level in skeletal muscle was inversely correlated with insulin sensitivity in Study A, analyzed with the WEB-based GEne SeT AnaLysis Toolkit (WebGestalt).

## Data Availability

The microarray data both studies have been deposited in the National Center for Biotechnology Information’s Gene Expression Omnibus (GEO) database (http://www.ncbi.nlm.nih.gov/geo); series accession number is GSE161721.

## Abbreviations

2DG: 2-Deoxy-D-glucose
ATGL: Adipocyte triglyceride lipase
BMI: Body mass index
COL1A1: Collagen type I alpha 1 chain
COL1A2: Collagen type I alpha 2 chain
COL3A1: Collagen type III alpha 1 chain
CPT1B: Carnitine palmitoyltransferase 1B
DG6P: 2-Deoxy-D-glucose 6-phosphate
ESRRA: Estrogen related receptor alpha
FABP3: Fatty acid binding protein 3
FBXW5: F-box and WD repeat domain containing 5
GCOS: GeneChip Operating System
GC-RMA: Guanine cytosine-content robust multi-array analysis
GEO: Gene Expression Omnibus
GO: Gene Ontology
HCR: histidine rich calcium binding protein
HOMA-IR: Homeostatic model assessment-insulin resistance
IMTG: Intramuscular triacylglycerol
IR: Insulin resistance
KLF5: Krüppel-like transcription factor
KRH: Krebs-Ringer-HEPES
LAMA4: Laminin subunit alpha 4
Limma: Linear models for microarray data
MAS5.0: Affymetrix microarray suite version 5.0
MEF2D: Myocyte enhancer factor 2D (MEF2D)
MLYCD: Malonyl-CoA decarboxylase
MSAT: Muscle SATellite cell
mtDNA: mitochondrial DNA content
mTOR: mammalian target-of-rapamycin
nDNA: nuclear DNA
OGTT: Oral glucose tolerance test
PLIER: Probe logarithmic intensity error estimation
PPARGC1A: Peroxisome proliferator-activated receptor gamma coactivator 1 alpha
QPCR: Quantitative real-time polymerase chain reaction
RMA: Robust multi-array analysis
SIRT2: Sirtuin 2
T2D: Type 2 diabetes
TEA: Triethanolamine
TSC2: TSC complex subunit 2
UCP2: Uncoupling protein 2
ULK1: Unc-51 like autophagy activating kinase 1
WebGestalt: WEB-based GEne SeT AnaLysis Toolkit
