# Supplementary material for: Relationship between insulin sensitivity and gene expression in human skeletal muscle
Source: BMC Endocr Disord. 2021 Feb 27;21:32. doi: 10.1186/s12902-021-00687-9 (PMC7912896; doi:10.1186/s12902-021-00687-9)
Supplement: Supplementary file 1 — Additional file 1: Supplementary Table S1. Clinical and biochemical characteristics of male subjects from study A. Supplementary Table S2. Clinical and biochemical characteristics of male subjects from study B. Supplementary Table S3. Genes of which expression levels in skeletal muscle were positively correlated with insulin sensitivity (1/HOMA-IR) in study A. Supplementary Table S4. Genes of which expression levels in skeletal muscle were inversely correlated with insulin sensitivity (1/HOMA-IR) in study A. Supplementary Table S5. Significantly enriched Gene Ontology (GO) categories in the 70 genes whose expression level in skeletal muscle positively correlated with insulin sensitivity in Study A, analyzed with the WEB-based GEne SeT AnaLysis Toolkit (WebGestalt). Supplementary Table S6. Significantly enriched Wikipathways, in the 70 genes whose expression level in skeletal muscle positively correlated with insulin sensitivity in Study A, analyzed with the WEB-based GEne SeT AnaLysis Toolkit (WebGestalt). Supplementary Table S7. Significantly enriched Gene Ontology (GO) categories in the 110 genes whose expression level in skeletal muscle was inversely correlated with insulin sensitivity in Study A, analyzed with the WEB-based GEne SeT AnaLysis Toolkit (WebGestalt). [file 12902_2021_687_MOESM1_ESM.docx]

**Supplementary Tables**

**Supplementary Table S1**: Clinical and biochemical characteristics of male subjects from study A.

N (family history of T2D) 38 (18)

Age (years) 37.71 ± 4.38

BMI (kg/m^2^) 28.47 ± 2.96

**OGTT plasma glucose (mmol/l)**

0 min 5.06 ± 0.55

120 min 5.88 ± 1.23

**OGTT plasma insulin (μU/ml)**

0 min 7.51 ± 3.21

**Surrogate measure of insulin sensitivity**

1/HOMA-IR 0.69 ± 0.25

Values are mean ± S.D.

**Abbreviations**: T2D, type 2 diabetes; BMI, body mass index; HOMA-IR, homeostasis model assessment of insulin resistance; OGTT, oral glucose tolerance test.

**Supplementary Table S2**: Clinical and biochemical characteristics of male subjects from study B.

N 9

Age (years) 25.33 ± 0.99

BMI (kg/m^2^) 24.57 ± 1.86

**Surrogate measure of insulin sensitivity**

1/HOMA-IR 1.17 ± 0.36

Values are mean ± S.D.

**Abbreviations**: BMI, body mass index; HOMA-IR, homeostasis model assessment of insulin resistance.

**Supplementary Table S3**: Genes of which expression levels in skeletal muscle were positively correlated with insulin sensitivity (1/HOMA-IR) in study A.

| **Gene Symbol** | **Entrez GeneID** | **GC-RMA** | | **PLIER** | | **RMA** | |
| --- | --- | --- | --- | --- | --- | --- | --- |
|  |  | **r** | ***P*** | **r** | ***P*** | **r** | ***P*** |
| *UCP2* | 7351 | 0.56 | < 0.001 | 0.61 | < 0.001 | 0.53 | 0.001 |
| *YPEL3* | 83719 | 0.53 | 0.001 | 0.45 | 0.006 | 0.53 | 0.001 |
| *ATG13* | 9776 | 0.53 | 0.001 | 0.42 | 0.012 | 0.48 | 0.003 |
| *ULK1* | 8408 | 0.51 | 0.002 | 0.43 | 0.010 | 0.59 | < 0.001 |
| *ZCCHC3* | 85364 | 0.51 | 0.002 | 0.38 | 0.024 | 0.42 | 0.013 |
| *DUSP14* | 11072 | 0.50 | 0.002 | 0.37 | 0.028 | 0.43 | 0.010 |
| *RGL2* | 5863 | 0.50 | 0.002 | 0.56 | < 0.001 | 0.48 | 0.004 |
| *UBE2R2* | 54926 | 0.51 | 0.002 | 0.38 | 0.023 | 0.50 | 0.002 |
| *CHMP6* | 79643 | 0.50 | 0.002 | 0.44 | 0.007 | 0.59 | < 0.001 |
| *SOX13* | 9580 | 0.51 | 0.002 | 0.46 | 0.006 | 0.49 | 0.003 |
| *KCNC4* | 3749 | 0.50 | 0.002 | 0.36 | 0.035 | 0.36 | 0.035 |
| *DIP2C* | 22982 | 0.49 | 0.003 | 0.45 | 0.007 | 0.50 | 0.002 |
| *TMEM94* | 9772 | 0.48 | 0.003 | 0.36 | 0.033 | 0.42 | 0.012 |
| *STRN4* | 29888 | 0.48 | 0.004 | 0.44 | 0.008 | 0.53 | 0.001 |
| *SUGP1* | 57794 | 0.48 | 0.004 | 0.37 | 0.029 | 0.47 | 0.005 |
| *CPT1B* | 1375 | 0.47 | 0.005 | 0.42 | 0.011 | 0.48 | 0.004 |
| *PITX3* | 5309 | 0.46 | 0.005 | 0.35 | 0.037 | 0.50 | 0.002 |
| *C16orf86* | 388284 | 0.46 | 0.005 | 0.43 | 0.010 | 0.46 | 0.005 |
| *UNKL* | 64718 | 0.46 | 0.005 | 0.47 | 0.004 | 0.44 | 0.008 |
| *UCKL1* | 54963 | 0.47 | 0.004 | 0.46 | 0.006 | 0.47 | 0.004 |
| *SUPT5H* | 6829 | 0.45 | 0.006 | 0.35 | 0.042 | 0.42 | 0.011 |
| *HRC* | 3270 | 0.46 | 0.006 | 0.43 | 0.009 | 0.43 | 0.011 |
| *PNPLA2* | 57104 | 0.45 | 0.007 | 0.37 | 0.028 | 0.47 | 0.005 |
| *ATOH8* | 84913 | 0.44 | 0.008 | 0.42 | 0.011 | 0.36 | 0.032 |
| *TSC2* | 7249 | 0.44 | 0.008 | 0.43 | 0.009 | 0.47 | 0.005 |
| *OBSCN* | 84033 | 0.44 | 0.008 | 0.44 | 0.008 | 0.48 | 0.004 |
| *HMOX1* | 3162 | 0.44 | 0.009 | 0.36 | 0.035 | 0.42 | 0.012 |
| *ARID1A* | 8289 | 0.43 | 0.009 | 0.36 | 0.031 | 0.40 | 0.016 |
| *FRS3* | 10817 | 0.43 | 0.010 | 0.42 | 0.012 | 0.46 | 0.005 |
| *SGCA* | 6442 | 0.43 | 0.010 | 0.38 | 0.023 | 0.40 | 0.017 |
| *MEF2D* | 4209 | 0.42 | 0.011 | 0.51 | 0.002 | 0.40 | 0.019 |
| *CIZ1* | 25792 | 0.42 | 0.011 | 0.42 | 0.013 | 0.48 | 0.003 |
| *CDC34* | 997 | 0.42 | 0.012 | 0.44 | 0.008 | 0.40 | 0.018 |
| *RALGDS* | 5900 | 0.42 | 0.012 | 0.43 | 0.010 | 0.53 | 0.001 |
| *UBALD2* | 283991 | 0.42 | 0.013 | 0.41 | 0.014 | 0.47 | 0.005 |
| *USF2* | 7392 | 0.41 | 0.013 | 0.43 | 0.010 | 0.38 | 0.023 |
| *HIGD2A* | 192286 | 0.42 | 0.013 | 0.34 | 0.043 | 0.36 | 0.033 |
| *SBF1* | 6305 | 0.41 | 0.014 | 0.39 | 0.021 | 0.35 | 0.039 |
| *WDTC1* | 23038 | 0.41 | 0.015 | 0.40 | 0.016 | 0.39 | 0.021 |
| *ATP6V0C* | 527 | 0.40 | 0.016 | 0.40 | 0.018 | 0.36 | 0.031 |
| *FCGRT* | 2217 | 0.40 | 0.017 | 0.37 | 0.030 | 0.39 | 0.021 |
| *ZNF503* | 84858 | 0.40 | 0.018 | 0.34 | 0.048 | 0.36 | 0.032 |
| *KIF1C* | 10749 | 0.39 | 0.019 | 0.42 | 0.013 | 0.37 | 0.029 |
| *DENND4B* | 9909 | 0.39 | 0.020 | 0.40 | 0.017 | 0.36 | 0.034 |
| *TFEB* | 7942 | 0.39 | 0.020 | 0.35 | 0.036 | 0.36 | 0.035 |
| *PLEC* | 5339 | 0.39 | 0.020 | 0.38 | 0.026 | 0.42 | 0.012 |
| *MRPL52* | 122704 | 0.39 | 0.021 | 0.38 | 0.023 | 0.41 | 0.015 |
| *SIRT2* | 22933 | 0.39 | 0.021 | 0.40 | 0.017 | 0.34 | 0.049 |
| *POU6F2* | 11281 | 0.39 | 0.022 | 0.41 | 0.015 | 0.40 | 0.017 |
| *DVL1* | 1855 | 0.38 | 0.023 | 0.34 | 0.045 | 0.37 | 0.031 |
| *TBC1D14* | 57533 | 0.38 | 0.023 | 0.37 | 0.030 | 0.40 | 0.017 |
| *RAB11FIP5* | 26056 | 0.38 | 0.023 | 0.37 | 0.030 | 0.35 | 0.039 |
| *ARFGAP2* | 84364 | 0.38 | 0.024 | 0.34 | 0.046 | 0.39 | 0.020 |
| *SPEN* | 23013 | 0.38 | 0.024 | 0.40 | 0.018 | 0.45 | 0.007 |
| *CASKIN2* | 57513 | 0.38 | 0.025 | 0.34 | 0.046 | 0.50 | 0.002 |
| *AKT1S1* | 84335 | 0.37 | 0.030 | 0.39 | 0.020 | 0.40 | 0.018 |
| *CAMK2A* | 815 | 0.37 | 0.030 | 0.38 | 0.025 | 0.35 | 0.042 |
| *FXYD1* | 5348 | 0.37 | 0.030 | 0.40 | 0.017 | 0.39 | 0.020 |
| *MPST* | 4357 | 0.36 | 0.031 | 0.40 | 0.018 | 0.43 | 0.010 |
| *FANCC* | 2176 | 0.36 | 0.033 | 0.43 | 0.011 | 0.42 | 0.013 |
| *SCGB1D2* | 10647 | 0.36 | 0.034 | 0.42 | 0.012 | 0.37 | 0.031 |
| *SQSTM1* | 8878 | 0.36 | 0.034 | 0.35 | 0.042 | 0.37 | 0.030 |
| *GET4* | 51608 | 0.36 | 0.035 | 0.40 | 0.016 | 0.45 | 0.006 |
| *SLC27A1* | 376497 | 0.35 | 0.037 | 0.39 | 0.021 | 0.43 | 0.010 |
| *DNAJB1* | 3337 | 0.35 | 0.039 | 0.37 | 0.030 | 0.40 | 0.018 |
| *FBXW5* | 54461 | 0.35 | 0.041 | 0.35 | 0.042 | 0.34 | 0.049 |
| *CALCOCO1* | 57658 | 0.35 | 0.042 | 0.34 | 0.046 | 0.35 | 0.040 |
| *JUND* | 3727 | 0.34 | 0.043 | 0.34 | 0.046 | 0.37 | 0.030 |
| *PLPP7* | 84814 | 0.34 | 0.043 | 0.38 | 0.025 | 0.35 | 0.038 |
| *PAIP2B* | 400961 | 0.34 | 0.045 | 0.50 | 0.002 | 0.40 | 0.018 |

**Abbreviations**: GC-RMA, GC-content robust multi-array average; PLIER, probe logarithmic intensity error; RMA, robust multi-array average; r, Spearman rank partial correlation coefficient.**Supplementary Table S4**: Genes of which expression levels in skeletal muscle were inversely correlated with insulin sensitivity (1/HOMA-IR) in study A.

| **Gene Symbol** | **Entrez GeneID** | **GC-RMA** | | **PLIER** | | **RMA** | |
| --- | --- | --- | --- | --- | --- | --- | --- |
|  |  | **r** | ***P*** | **r** | ***P*** | **r** | ***P*** |
| *P4HA2* | 8974 | -0.64 | < 0.001 | -0.60 | < 0.001 | -0.70 | < 0.001 |
| *KDELR3* | 11015 | -0.57 | < 0.001 | -0.40 | 0.016 | -0.42 | 0.012 |
| *HSD17B12* | 51144 | -0.53 | 0.001 | -0.51 | 0.002 | -0.50 | 0.002 |
| *CGGBP1* | 8545 | -0.53 | 0.001 | -0.46 | 0.005 | -0.40 | 0.017 |
| *CINP* | 51550 | -0.53 | 0.001 | -0.44 | 0.008 | -0.58 | < 0.001 |
| *TMEM128* | 85013 | -0.53 | 0.001 | -0.40 | 0.018 | -0.44 | 0.009 |
| *NAE1* | 8883 | -0.53 | 0.001 | -0.48 | 0.004 | -0.56 | < 0.001 |
| *LAMA4* | 3910 | -0.52 | 0.001 | -0.54 | 0.001 | -0.53 | 0.001 |
| *COL1A1* | 1277 | -0.51 | 0.002 | -0.52 | 0.001 | -0.51 | 0.002 |
| *STK38* | 11329 | -0.50 | 0.002 | -0.46 | 0.005 | -0.37 | 0.027 |
| *DYNC1LI1* | 51143 | -0.50 | 0.002 | -0.41 | 0.014 | -0.44 | 0.008 |
| *GASK1B* | 51313 | -0.52 | 0.002 | -0.49 | 0.003 | -0.46 | 0.006 |
| *ENPP5* | 59084 | -0.49 | 0.003 | -0.43 | 0.009 | -0.44 | 0.008 |
| *CTPS1* | 1503 | -0.48 | 0.003 | -0.35 | 0.038 | -0.36 | 0.032 |
| *TMEM100* | 55273 | -0.48 | 0.003 | -0.53 | 0.001 | -0.47 | 0.004 |
| *APC* | 324 | -0.48 | 0.004 | -0.46 | 0.005 | -0.42 | 0.011 |
| *XK* | 7504 | -0.47 | 0.004 | -0.48 | 0.003 | -0.42 | 0.011 |
| *COL1A2* | 1278 | -0.47 | 0.004 | -0.47 | 0.004 | -0.46 | 0.005 |
| *BUD31* | 8896 | -0.46 | 0.005 | -0.49 | 0.003 | -0.36 | 0.031 |
| *MRI1* | 84245 | -0.46 | 0.006 | -0.47 | 0.004 | -0.47 | 0.004 |
| *ENPP4* | 22875 | -0.45 | 0.006 | -0.39 | 0.022 | -0.42 | 0.011 |
| *COL3A1* | 1281 | -0.46 | 0.006 | -0.46 | 0.005 | -0.42 | 0.012 |
| *TIMM8B* | 26521 | -0.45 | 0.007 | -0.37 | 0.027 | -0.46 | 0.005 |
| *NDUFC2* | 4718 | -0.45 | 0.007 | -0.37 | 0.029 | -0.35 | 0.039 |
| *TAF9* | 6880 | -0.45 | 0.007 | -0.42 | 0.013 | -0.35 | 0.037 |
| *KIF21A* | 55605 | -0.45 | 0.007 | -0.43 | 0.011 | -0.45 | 0.007 |
| *GTF2E1* | 2960 | -0.44 | 0.008 | -0.53 | 0.001 | -0.38 | 0.026 |
| *TRIM44* | 54765 | -0.44 | 0.008 | -0.39 | 0.021 | -0.34 | 0.049 |
| *C4orf3* | 401152 | -0.43 | 0.009 | -0.45 | 0.007 | -0.46 | 0.005 |
| *SPTSSA* | 171546 | -0.43 | 0.009 | -0.49 | 0.003 | -0.48 | 0.004 |
| *SPARC* | 6678 | -0.44 | 0.009 | -0.42 | 0.013 | -0.39 | 0.020 |
| *LEO1* | 123169 | -0.43 | 0.010 | -0.48 | 0.003 | -0.43 | 0.010 |
| *IDH1* | 3417 | -0.43 | 0.010 | -0.44 | 0.008 | -0.43 | 0.009 |
| *DDX50* | 79009 | -0.43 | 0.010 | -0.38 | 0.023 | -0.38 | 0.026 |
| *TDP2* | 51567 | -0.43 | 0.011 | -0.44 | 0.008 | -0.40 | 0.016 |
| *UBE2F* | 140739 | -0.42 | 0.011 | -0.45 | 0.007 | -0.42 | 0.012 |
| *MFSD1* | 64747 | -0.42 | 0.011 | -0.41 | 0.015 | -0.50 | 0.002 |
| *QDPR* | 5860 | -0.42 | 0.012 | -0.36 | 0.031 | -0.38 | 0.023 |
| *POLB* | 5423 | -0.42 | 0.012 | -0.41 | 0.016 | -0.47 | 0.004 |
| *RAP1A* | 5906 | -0.41 | 0.013 | -0.34 | 0.042 | -0.41 | 0.016 |
| *KPNA5* | 3841 | -0.41 | 0.014 | -0.51 | 0.002 | -0.38 | 0.025 |
| *JMJD1C* | 221037 | -0.41 | 0.014 | -0.40 | 0.016 | -0.42 | 0.012 |
| *SLIT2* | 9353 | -0.41 | 0.014 | -0.47 | 0.005 | -0.39 | 0.020 |
| *LAP3* | 51056 | -0.41 | 0.015 | -0.38 | 0.026 | -0.37 | 0.028 |
| *HS3ST5* | 222537 | -0.41 | 0.015 | -0.37 | 0.029 | -0.38 | 0.024 |
| *RBX1* | 9978 | -0.40 | 0.016 | -0.34 | 0.045 | -0.35 | 0.038 |
| *CCPG1* | 9236 | -0.40 | 0.016 | -0.46 | 0.005 | -0.41 | 0.013 |
| *SNX21* | 90203 | -0.41 | 0.016 | -0.44 | 0.008 | -0.45 | 0.007 |
| *AP3M1* | 26985 | -0.40 | 0.017 | -0.40 | 0.016 | -0.45 | 0.006 |
| *VPS13C* | 54832 | -0.40 | 0.017 | -0.41 | 0.015 | -0.36 | 0.032 |
| *TUBA1A* | 7846 | -0.40 | 0.018 | -0.40 | 0.018 | -0.38 | 0.026 |
| *PSMA1* | 5682 | -0.40 | 0.018 | -0.36 | 0.035 | -0.33 | 0.049 |
| *RNF141* | 50862 | -0.39 | 0.019 | -0.36 | 0.034 | -0.37 | 0.031 |
| *COPB2* | 9276 | -0.39 | 0.019 | -0.49 | 0.003 | -0.37 | 0.027 |
| *FAHD1* | 81889 | -0.39 | 0.022 | -0.39 | 0.021 | -0.39 | 0.019 |
| *LRRFIP2* | 9209 | -0.38 | 0.024 | -0.41 | 0.014 | -0.38 | 0.023 |
| *KIAA0408* | 9729 | -0.38 | 0.024 | -0.34 | 0.043 | -0.43 | 0.010 |
| *SH3GLB1* | 51100 | -0.38 | 0.024 | -0.42 | 0.012 | -0.34 | 0.048 |
| *EIF2S2* | 8894 | -0.38 | 0.024 | -0.38 | 0.022 | -0.38 | 0.025 |
| *LZIC* | 84328 | -0.38 | 0.024 | -0.35 | 0.036 | -0.42 | 0.011 |
| *C7orf25* | 79020 | -0.38 | 0.024 | -0.34 | 0.047 | -0.35 | 0.040 |
| *TRIP4* | 9325 | -0.38 | 0.025 | -0.48 | 0.003 | -0.46 | 0.005 |
| *UBE2V2* | 7336 | -0.38 | 0.025 | -0.35 | 0.037 | -0.40 | 0.018 |
| *DERA* | 51071 | -0.38 | 0.025 | -0.37 | 0.030 | -0.37 | 0.028 |
| *RANBP2* | 5903 | -0.38 | 0.025 | -0.36 | 0.035 | -0.35 | 0.042 |
| *CKAP2* | 26586 | -0.38 | 0.025 | -0.45 | 0.007 | -0.43 | 0.010 |
| *TAF2* | 6873 | -0.38 | 0.026 | -0.34 | 0.048 | -0.37 | 0.029 |
| *DLK1* | 8788 | -0.37 | 0.027 | -0.40 | 0.018 | -0.35 | 0.037 |
| *MYL6* | 4637 | -0.37 | 0.027 | -0.44 | 0.008 | -0.37 | 0.027 |
| *L3HYPDH* | 112849 | -0.37 | 0.027 | -0.44 | 0.008 | -0.43 | 0.010 |
| *UCHL5* | 51377 | -0.37 | 0.027 | -0.43 | 0.010 | -0.40 | 0.017 |
| *OSBPL11* | 114885 | -0.37 | 0.027 | -0.35 | 0.037 | -0.40 | 0.018 |
| *ARHGAP18* | 93663 | -0.37 | 0.028 | -0.38 | 0.023 | -0.38 | 0.023 |
| *CAPRIN2* | 65981 | -0.37 | 0.028 | -0.41 | 0.014 | -0.57 | 0 |
| *AGO3* | 192669 | -0.37 | 0.030 | -0.43 | 0.009 | -0.34 | 0.044 |
| *FYTTD1* | 84248 | -0.37 | 0.030 | -0.38 | 0.022 | -0.37 | 0.027 |
| *UFM1* | 51569 | -0.37 | 0.030 | -0.36 | 0.036 | -0.42 | 0.011 |
| *LRRTM4* | 80059 | -0.37 | 0.030 | -0.37 | 0.029 | -0.45 | 0.007 |
| *GBE1* | 2632 | -0.37 | 0.030 | -0.35 | 0.037 | -0.36 | 0.035 |
| *NUDCD1* | 84955 | -0.37 | 0.031 | -0.34 | 0.045 | -0.38 | 0.025 |
| *COX11* | 1353 | -0.36 | 0.031 | -0.37 | 0.029 | -0.36 | 0.035 |
| *PMEPA1* | 56937 | -0.37 | 0.031 | -0.43 | 0.010 | -0.34 | 0.045 |
| *MRPL42* | 28977 | -0.37 | 0.031 | -0.40 | 0.018 | -0.43 | 0.011 |
| *PTRHD1* | 391356 | -0.36 | 0.032 | -0.34 | 0.047 | -0.39 | 0.019 |
| *MTERF1* | 7978 | -0.36 | 0.032 | -0.41 | 0.014 | -0.44 | 0.009 |
| *S100A8* | 6279 | -0.36 | 0.033 | -0.37 | 0.028 | -0.34 | 0.048 |
| *UTP6* | 55813 | -0.36 | 0.033 | -0.42 | 0.012 | -0.37 | 0.027 |
| *SAMD9L* | 219285 | -0.36 | 0.033 | -0.38 | 0.025 | -0.42 | 0.012 |
| *KIAA0586* | 9786 | -0.36 | 0.035 | -0.33 | 0.050 | -0.36 | 0.036 |
| *PSMD6* | 9861 | -0.36 | 0.036 | -0.38 | 0.024 | -0.47 | 0.004 |
| *ADH5* | 128 | -0.36 | 0.036 | -0.42 | 0.012 | -0.37 | 0.028 |
| *TMEM165* | 55858 | -0.36 | 0.036 | -0.45 | 0.007 | -0.42 | 0.011 |
| *SACS* | 26278 | -0.35 | 0.038 | -0.34 | 0.047 | -0.36 | 0.032 |
| *OAZ1* | 4946 | -0.35 | 0.038 | -0.39 | 0.022 | -0.34 | 0.047 |
| *FSTL1* | 11167 | -0.35 | 0.038 | -0.37 | 0.026 | -0.41 | 0.014 |
| *INTS2* | 57508 | -0.35 | 0.039 | -0.37 | 0.027 | -0.44 | 0.008 |
| *RABGGTB* | 5876 | -0.35 | 0.039 | -0.42 | 0.011 | -0.41 | 0.015 |
| *FERMT2* | 10979 | -0.35 | 0.039 | -0.36 | 0.035 | -0.34 | 0.048 |
| *RRP15* | 51018 | -0.35 | 0.039 | -0.41 | 0.015 | -0.39 | 0.019 |
| *CYBRD1* | 79901 | -0.35 | 0.041 | -0.36 | 0.032 | -0.37 | 0.029 |
| *ATP6V0E1* | 8992 | -0.35 | 0.041 | -0.34 | 0.044 | -0.37 | 0.027 |
| *PHF10* | 55274 | -0.35 | 0.041 | -0.33 | 0.049 | -0.37 | 0.030 |
| *MRPL27* | 51264 | -0.35 | 0.041 | -0.35 | 0.038 | -0.33 | 0.050 |
| *VPS33B* | 26276 | -0.34 | 0.044 | -0.38 | 0.023 | -0.38 | 0.024 |
| *ABITRAM* | 54942 | -0.34 | 0.044 | -0.37 | 0.030 | -0.41 | 0.016 |
| *IDI2-AS1* | 55853 | -0.34 | 0.046 | -0.37 | 0.031 | -0.35 | 0.040 |
| *NID2* | 22795 | -0.34 | 0.046 | -0.39 | 0.021 | -0.35 | 0.042 |
| *MCTS1* | 28985 | -0.34 | 0.046 | -0.37 | 0.031 | -0.40 | 0.018 |
| *ZNF613* | 79898 | -0.34 | 0.047 | -0.43 | 0.009 | -0.35 | 0.038 |
| *STK39* | 27347 | -0.33 | 0.049 | -0.40 | 0.019 | -0.39 | 0.020 |

**Abbreviations**: GC-RMA, GC-content robust multi-array average; PLIER, probe logarithmic intensity error; RMA, robust multi-array average; r, Spearman rank partial correlation coefficient.

**Supplementary Table S5**: Significantly enriched Gene Ontology (GO) categories in the 70 genes whose expression level in skeletal muscle positively correlated with insulin sensitivity in Study A, analyzed with the WEB-based GEne SeT AnaLysis Toolkit (WebGestalt).

Significantly enriched categories under the sub-root biological process:

| **Macroautophagy** | **GO:0016236** |
| --- | --- |
| Gene Set Size=286; Overlap Genes=9; Expected Value=1.05; Enrichment Ratio=8.60; *P*=9.04x10^-7^; FDR=0.003 | |

| **Autophagy** | **GO:0006914** |
| --- | --- |
| Gene Set Size=473; Overlap Genes=11; Expected Value=1.73; Enrichment Ratio=6.35; *P*=9.95x10^-7^; FDR=0.003 | |

| **Process utilizing autophagic mechanism** | **GO:0061919** |
| --- | --- |
| Gene Set Size=473; Overlap Genes=11; Expected Value=1.73; Enrichment Ratio=6.35; *P*=9.95x10^-7^; FDR=0.003 | |

| **Regulation of autophagy** | **GO:0010506** |
| --- | --- |
| Gene Set Size=304; Overlap Genes=9; Expected Value=1.11; Enrichment Ratio=8.09; *P*=1.49x10^-6^; FDR=0.003 | |

| **Regulation of macroautophagy** | **GO:0016241** |
| --- | --- |
| Gene Set Size=160; Overlap Genes=7; Expected Value=0.59; Enrichment Ratio=11.95; *P*=1.86x10^-6^; FDR=0.003 | |

Significantly enriched categories under the sub-root molecular function:

| **GTPase binding** | **GO:0051020** |
| --- | --- |
| Gene Set Size=646; Overlap Genes=12; Expected Value=2.25; Enrichment Ratio=5.34; *P*=1.18x10^-6^; FDR=0.002 | |

| **small GTPase binding** | **GO:0031267** |
| --- | --- |
| Gene Set Size=551; Overlap Genes=11; Expected Value=1.92; Enrichment Ratio=5.74; *P*=2.59x10^-6^; FDR=0.002 | |

| **Ras GTPase binding** | **GO:0017016** |
| --- | --- |
| Gene Set Size=534; Overlap Genes=10; Expected Value=1.86; Enrichment Ratio=5.38; *P*=1.37x10^-5^; FDR=0.009 | |

The first row lists the category name and corresponding GO ID under the sub-root biological process and molecular function. FDR, False Discovery Rate.

**Supplementary Table S6**: Significantly enriched Wikipathways, in the 70 genes whose expression level in skeletal muscle positively correlated with insulin sensitivity in Study A, analyzed with the WEB-based GEne SeT AnaLysis Toolkit (WebGestalt).

| **PI3K-AKT-mTOR signaling pathway and therapeutic opportunities (WP3844)** |
| --- |
| Gene Set Size=30; Overlap Genes=4; Expected Value=0.14; Enrichment Ratio=27.73; *P*=1.12x10^-5^; FDR=0.006 |

| **Neurodegeneration with brain iron accumulation (NBIA) subtypes pathway (WP4577)** |
| --- |
| Gene Set Size=44; Overlap Genes=4; Expected Value=0.21; Enrichment Ratio=18.91; *P*=5.28x10^-5^; FDR=0.014 |

| **Thermogenesis (WP4321)** |
| --- |
| Gene Set Size=108; Overlap Genes=5; Expected Value=0.52; Enrichment Ratio=9.63; *P*=1.48x10^-4^; FDR=0.026 |

The first row lists the pathway name and corresponding ID. FDR, False Discovery Rate.

**Supplementary Table S7**: Significantly enriched Gene Ontology (GO) categories in the 110 genes whose expression level in skeletal muscle was inversely correlated with insulin sensitivity in Study A, analyzed with the WEB-based GEne SeT AnaLysis Toolkit (WebGestalt).

Significantly enriched categories under the sub-root molecular function:

| **Platelet-derived growth factor binding** | **GO:0048407** |
| --- | --- |
| Gene Set Size=11; Overlap Genes=3; Expected Value=0.06; Enrichment Ratio=51.67; *P*=2.27x10^-5^; FDR=0.04 | |

Significantly enriched categories under the sub-root cellular component:

| **Fibrillar collagen trimer** | **GO:0005583** |
| --- | --- |
| Gene Set Size=11; Overlap Genes=3; Expected Value=0.05; Enrichment Ratio=58.52; *P*=1.57x10^-5^; FDR=0.009 | |

| **Banded collagen fibril** | **GO:0098643** |
| --- | --- |
| Gene Set Size=11; Overlap Genes=3; Expected Value=0.05; Enrichment Ratio=58.52; *P*=1.57x10^-5^; FDR=0.009 | |

| **Complex of collagen trimers** | **GO:0098644** |
| --- | --- |
| Gene Set Size=19; Overlap Genes=3; Expected Value=0.09; Enrichment Ratio=33.88; *P*=8.95x10^-5^; FDR=0.04 | |

The first row lists the category name and corresponding GO ID under the sub-roots molecular function and cellular component. FDR, False Discovery Rate.
